# A new metabolic trait in an acetogen: Mixed acid fermentation of fructose in a methylene‐tetrahydrofolate reductase mutant of *Acetobacterium woodii*

**DOI:** 10.1111/1758-2229.13160

**Published:** 2023-05-07

**Authors:** Jimyung Moon, Anja Schubert, Anja Poehlein, Rolf Daniel, Volker Müller

**Affiliations:** ^1^ Department of Molecular Microbiology & Bioenergetics, Institute of Molecular Biosciences Johann Wolfgang Goethe University Frankfurt Germany; ^2^ Göttingen Genomics Laboratory, Institute for Microbiology and Genetics Georg August University Göttingen Germany

## Abstract

To inactivate the Wood–Ljungdahl pathway in the acetogenic model bacterium *Acetobacterium woodii*, the genes *metVF* encoding two of the subunits of the methylene‐tetrahydrofolate reductase were deleted. As expected, the mutant did not grow on C1 compounds and also not on lactate, ethanol or butanediol. In contrast to a mutant in which the first enzyme of the pathway (hydrogen‐dependent CO_2_ reductase) had been genetically deleted, cells were able to grow on fructose, albeit with lower rates and yields than the wild‐type. Growth was restored by addition of an external electron sink, glycine betaine + CO_2_ or caffeate. Resting cells pre‐grown on fructose plus an external electron acceptor fermented fructose to two acetate and four hydrogen, that is, performed hydrogenogenesis. Cells pre‐grown under fermentative conditions on fructose alone redirected carbon and electrons to form lactate, formate, ethanol as well as hydrogen. Apparently, growth on fructose alone induced enzymes for mixed acid fermentation (MAF). Transcriptome analyses revealed enzymes potentially involved in MAF and a quantitative model for MAF from fructose in *A. woodii* is presented.

## INTRODUCTION

The Wood–Ljungdahl pathway (WLP) is one of the seven known pathways for the fixation of carbon dioxide found in nature (Garritano et al., 2022). It is present in strictly anaerobic microorganisms such as methanogenic archaea, sulphate reducing and acetogenic bacteria and catalyses the anabolic formation of acetyl‐CoA from two molecules of CO_2_ (Schauder et al., 1988; Wolfe, 1993; Wood et al., 1986). In acetogens, the WLP is also involved in catabolism and acetyl‐CoA is further converted via acetyl phosphate to acetate, the characteristic and name‐giving end product of CO_2_ reduction in this group of bacteria (Drake, 1994; Müller, 2003). The pathway is linear with two branches. In the carbonyl branch, one molecule of CO_2_ is first reduced to carbon monoxide that is bound to the central enzyme of the pathway, the CO dehydrogenase/acetyl‐CoA synthase (CODH/ACS; Pezacka & Wood, 1984; Raybuck et al., 1988). In the methyl branch, one molecule of CO_2_ is first reduced to formate by a formate dehydrogenase; in the model species *Acetobacterium woodii* and *Thermoanaerobacter kivui*, hydrogen is the electron donor for CO_2_ reduction and hence, the enzyme was named hydrogen‐dependent CO_2_ reductase (HDCR; Dietrich et al., 2022; Schuchmann & Müller, 2013; Schwarz et al., 2018). Formate is then bound to the C1 carrier tetrahydrofolate (THF) giving formyl‐THF (Himes & Harmony, 1973; Lovell et al., 1988), water is split off, and the resulting methenyl‐THF is reduced via methylene‐THF to methyl‐THF (Bertsch et al., 2015; Ragsdale & Ljungdahl, 1984). The methyl group is then transferred to the CODH/ACS and combined with CO and CoA to acetyl‐CoA (Ragsdale, 2008).

The WLP enables acetogens to grow lithotrophically on H_2_ + CO_2_ according to:

(1) $${\text{4H}}_{2}+{\text{2CO}}_{2}\to {\mathrm{CH}}_{3}\text{COOH}+{\text{2H}}_{2}O\;\text{∆}{G_{0}}^{\prime }=-95\;\mathrm{kJ}/\mathrm{mol}.$$

The WLP also enables acetogens to grow on other C1 substrates that are intermediates of the pathway such as formate (Moon et al., 2021), CO (Diekert & Thauer, 1978; Diender et al., 2015; Genthner & Bryant, 1982; Savage et al., 1987; Weghoff & Müller, 2016) or methyl groups (Bache & Pfennig, 1981; Kremp et al., 2018; Kremp & Müller, 2021; Lechtenfeld et al., 2018; Litty et al., 2022; Stupperich & Konle, 1993; van der Meijden et al., 1984).

Acetogens are metabolically very versatile and can also grow heterotrophically on sugars, carboxylic acids and aldehydes (Bache & Pfennig, 1981; Drake et al., 2008; Gößner et al., 1994; Moon et al., 2019; Seifritz et al., 1999; Trifunovic et al., 2020; Weghoff et al., 2015). Actually, the WLP was discovered during glucose fermentation in *Moorella thermoacetica* (formerly *Clostridium thermoaceticum*; Fontaine et al., 1942). This bacterium oxidises glucose via the Embden–Meyerhoff–Parnas pathway to pyruvate and then further to 2 moles of acetate, 2 moles of CO_2_, and 8 electrons are generated (Drake & Daniel, 2004). In fermentation balances, no other product but a third mole of acetate had been found, and later it was demonstrated that 2 moles of CO_2_ were reduced with the eight electrons to a third mole of acetate by a novel pathway, the WLP (Fontaine et al., 1942). This type of fermentation was called homoacetogenesis and proceeds in two steps:

(2) $$\begin{array}{l} C_{6}H_{12}O_{6}+{\text{2H}}_{2}O\\ \;\to {\text{2CH}}_{3}\text{COOH}+{\text{2CO}}_{2}+{\text{4H}}_{2}\;\text{∆}{G_{0}}^{\prime }=-206.3\;\mathrm{kJ}/\mathrm{mol}, \end{array}$$

(3) $${\text{2CO}}_{2}+{\text{4H}}_{2}\to {\mathrm{CH}}_{3}\text{COOH}+{\text{2H}}_{2}O\;\text{∆}{G_{0}}^{\prime }=-95\;\mathrm{kJ}/\mathrm{mol},$$

(4) $$C_{6}H_{12}O_{6}\to {\text{3CH}}_{3}\text{COOH}\;\text{∆}{G_{0}}^{\prime }=-301.3\;\mathrm{kJ}/\mathrm{mol}.$$

The reduction of CO_2_ with electrons derived from molecular hydrogen conserves energy in the form of ATP by a respiratory mechanism, but in *A. woodii* only 0.3 ATP per mol of acetate are formed (Hess, Schuchmann, & Müller, 2013; Matthies et al., 2014); this is only 7.5% of the ATP gain of glycolysis plus pyruvate oxidation. The energetic benefit of using the WLP clearly is that all the acetyl‐CoA produced from glucose by glycolysis and pyruvate oxidation can be used for energy‐conserving acetate formation. This allows the bacteria to produce the maximum amount of ATP that can be obtained by glycolysis and pyruvate oxidation, 4 ATP/mol glucose.

Glucose oxidation to acetate, CO_2_ and hydrogen (Equation 5) gives the highest possible ATP yield for a fermentation but is performed only by a few bacteria such as, for example *Thermotoga maritima*, in pure culture (Schröder et al., 1994):

(5) $$\begin{array}{l} C_{6}H_{12}O_{6}+{\text{2H}}_{2}O\\ \;\to {\text{2CH}}_{3}\text{COOH}+{\text{2CO}}_{2}+{\text{4H}}_{2}\;\text{∆}G^{\prime }=-290\;\mathrm{kJ}/\mathrm{mol}\;\mathrm{at}\;80{}^{\circ}C. \end{array}$$

Oxidation of NADH coupled to reduction of protons to hydrogen is highly endergonic and only bacteria that have an electron‐bifurcating hydrogenase can overcome this steep energetic barrier (Schut & Adams, 2009). Such an enzyme is also present in *A. woodii* (Schuchmann & Müller, 2012; Wiechmann et al., 2020), but fermentation of glucose according to Equation 5 has never been observed in pure cultures. In contrast, if the hydrogen concentration is kept low by a syntrophic, methanogenic partner, *A. woodii* converted fructose according to Equation 5 and the hydrogen and CO_2_ produced were used by the syntrophic methanogenic partner to produce methane, demonstrating the presence of the enzymatic machinery to catalyse this fermentation (Winter & Wolfe, 1980).

Electrons could also be disposed by reducing an intermediate of the pathway, that is, by classical fermentation. If intermediates of the pathway such as acetyl‐CoA or pyruvate would have to act as electron acceptor, less acetate could be formed and for any acetate not produced, one ATP is lost. Nevertheless, a mixed acid fermentation (MAF) is well known, for example, in *Escherichia coli* (Xu et al., 1999), but this has never been observed in *A. woodii* although this model acetogen has all the enzymes required for a MAF, that is, CoA‐dependent acetaldehyde and alcohol dehydrogenases, lactate dehydrogenases, pyruvate:formate lyase and formate dehydrogenases (Poehlein et al., 2012).

The strict requirement of the WLP for glucose/fructose oxidation and/or removal of hydrogen by *T. kivui* and *A. woodii* were demonstrated recently by a genetic approach. HDCR deletion mutants could no longer convert H_2_ + CO_2_ to formate, the first step in the WLP, as expected. But they also did not grow on glucose/fructose anymore (Jain et al., 2020; Moon et al., 2023). We speculated that growth on hexoses may be restored by allowing the cells to recapture hydrogen and thus have attempted to genetically delete an enzyme downstream of the HDCR, the methylene‐THF reductase. This mutant could be generated, and we discovered that the mutant performs so far unknown fermentative metabolism during growth on fructose.

## EXPERIMENTAL PROCEDURES

### *Cultivation of* A. woodii

*A. woodii* strains DSM1030 (wild‐type), ∆*pyrE* or ∆*metVF* were cultivated under anoxic conditions at 30°C in bicarbonate‐buffered complex medium described previously (Heise et al., 1989). As substrates for growth, 20 mM of fructose or 20 mM of fructose + 80 mM of glycine betaine were used. Growth was monitored by measuring the optical density at 600 nm (OD_600_).

### *Generation of* A. woodii *ΔmetVF mutant*

For the generation of a Δ*metVF* mutant, the plasmid pMTL84151_AW_dmetVF was constructed in *E. coli* HB101 (Promega, Madison, WI) and transformed into *A. woodii* Δ*pyrE* strain, as described previously (Westphal et al., 2018). The plasmid was modified from pMTL84151 (Heap et al., 2009) as suicide plasmid which lacks a Gram‐positive replicon. In pMTL84151_AW_dmetVF, 450 bp of upstream flanking regions (UFR) of *metV* (Awo_c09300) and 700 bp of downstream flanking regions (DFR) of *metF* (Awo_c09310) were cloned into the multiple cloning sites for deletion of the *metVF* genes via homologous recombination. Moreover, this plasmid contains a *catP* marker for chloramphenicol/thiamphenicol resistance from *Clostridium perfringens* (Werner et al., 1977) and a heterologous *pyrE* from *Eubacterium limosum* (Wiechmann et al., 2020) as a counter selectable marker. The first selection was achieved on an agar plate with complex medium supplemented with 20 mM of fructose and 30 ng/μL of thiamphenicol after transformation of pMTL84151_AW_dmetVF into *A. woodii* Δ*pyrE* strain by electroporation (625 V, 25 μF, 600 Ω, in 1 mm cuvettes). The second selection was carried out on an agar plate with minimal medium (Westphal et al., 2018) supplemented with 20 mM of fructose, 1 μg/mL of uracil and 1 mg/mL of 5‐FOA. The deleted region was analysed by PCR with oligonucleotides which bind in front of UFR and behind DFR of the respective gene: aus_metVF_for (5′‐ATGATTGCTGATGAAAGAGGATTTTT‐3′) and aus_metVF_rev (5′‐AAGTCCCGCCAAGTTCATC‐3′). To check the purity of the mutant, oligonucleotides which bind in *metV* and *metF* genes were used for PCR: in_metVF_for (5′‐GAGTGTTACCTGAATGGGACC‐3′) and in_metVF_rev (5′‐CGTCTGACTGTAAGGCAATACG‐3′). The deleted region of the mutant was further verified by Sanger sequencing (Sanger et al., 1977).

### Transcriptome analyses

For transcriptome analyses, the wild‐type and the ∆*metVF* mutant were grown on 20 mM of fructose and harvested in the exponential growth phase at an OD_600_ of 0.2. RNA preparation and RNA sequencing were carried out as described previously (Moon et al., 2021). Subsequently, gene expression levels of the ∆*metVF* mutant were compared to those of the wild‐type. Genes with a log_2_‐fold change of transcript levels of +2/−2 and a *p*‐adjust value <0.05 were considered differentially expressed.

### Preparation of resting cells

Cells of the Δ*metVF* mutant were grown either on 20 mM of fructose or 20 mM of fructose + 80 mM of glycine betaine in 0.5–3 L of bicarbonate‐buffered complex medium to late exponential growth phase (on 20 mM of fructose, OD_600_ of 0.25; on 20 mM of fructose +80 mM of glycine betaine, OD_600_ of 1.5). After harvest by centrifugation (Avanti™J‐25 and JA‐10 Fixed‐Angle Rotor; Beckman Coulter, Brea, CA) at 8000 rpm and 4°C for 10 min, cells were washed with 30 mL of buffer containing 50 mM of imidazole (pH 7.0), 20 mM of KCl, 20 mM of MgSO_4_, 4 mM of DTE and 4 μM of resazurin and pelleted by centrifugation at 8500 rpm and 4°C for 10 min (Avanti™J‐25 and JA‐25.50 Fixed‐Angle Rotor; Beckman Coulter, Brea, CA). Subsequently, the cells were resuspended in 5 mL of imidazole buffer and transferred to 16‐mL Hungate tubes. All steps were performed under strictly anoxic conditions in an anoxic chamber (Coy Laboratory Products, Grass Lake, MI) filled with N_2_/H_2_ (96%–98%/2%–4%; v/v). The gas phase of the cell suspensions was changed to 100% N_2_ to remove residual H_2_ from the anoxic chamber. The total protein concentration of the resting cells was determined according to Schmidt et al. (1963).

### Cell suspension experiments

The cells were resuspended in 10 mL of imidazole buffer (50 mM of imidazole, 20 mM of KCl, 20 mM of NaCl, 20 mM of MgSO_4_, 60 mM of KHCO_3_, 4 mM of DTE, 4 μM of resazurin, pH 7.0) in 120 mL serum flasks to a final protein concentration of 2 mg/mL under an atmosphere of N_2_/CO_2_ (80:20, v/v). 20 mM of fructose was given as carbon and energy source, and if necessary, 80 mM of glycine betaine, 4 mM of caffeate or H_2_ (100%, 1 bar) was added to the resting cells. The cells were pre‐incubated at 30°C in water bath with shaking (150 rpm) and the experiments were started by the addition of the substrate(s). One millilitre of samples were taken at each time point for determination of metabolites.

### Chemical analysis

H_2_ was determined by gas chromatography as described previously (Weghoff & Müller, 2016). The concentrations of ethanol, acetoin and acetate were determined by gas chromatography as described previously (Trifunović et al., 2016). The concentrations of fructose, formate and lactate were determined by high‐performance liquid chromatography (Moon et al., 2019). Alanine was determined enzymatically using alanine dehydrogenase as described previously (Dönig & Müller, 2018). Glycerol and dihydroxyacetone were measured enzymatically (Trifunovic et al., 2021). Caffeate was determined photometrically at 312 nm using an extinction coefficient of 13.72 mM^−1^ cm^−1^ (Dilling et al., 2007).

## RESULTS

### *Deletion of the metVF genes of* A. woodii

**FIGURE 1:**
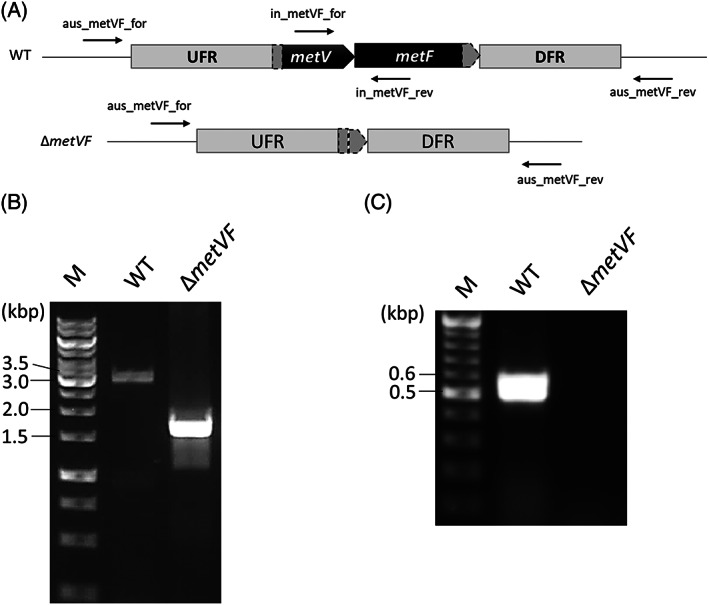
Deletion of the *metVF* genes in the chromosome of *Acetobacterium woodii*. (A) Only 42 bp of the *metV* gene and 36 bp of the *metF* gene remained in the Δ*metVF* mutant. (B) Genotypic analyses of the Δ*metVF* mutant were performed using colony PCR with primers binding outside the deleted region (aus_metVF_for and aus_metVF_rev) or inside (C) (in_metVF_for and in_metVF_rev). DFR, downstream flanking regions; UFR, upstream flanking regions.

The MTHFR of *A. woodii* consists of the three subunits RnfC2, MetV and MetF (Bertsch et al., 2015), encoded by *rnfC2* (Awo_c09290), *metV* (Awo_c09300) and *metF* (Awo_c09310) that are clustered with other genes encoding the enzymes of the methyl branch of the WLP, such as *fhs1* (Awo_c09260; formyl‐THF synthetase), *fchA* (Awo_c09270; methenyl‐THF cyclohydrolase) and *folD* (Awo_c09280; methylene‐THF dehydrogenase; Poehlein et al., 2012). We deleted the *metVF* genes through allelic exchange mutagenesis leaving only 42 bp including the start codon of *metV* and 36 bp including the stop codon of *metF* (Figure 1A). PCR experiments with primers binding outside the deleted region showed that the *metVF* genes were successfully deleted (Figure 1B). Consequently, the *metV* and *metF* genes could not be amplified with primers binding inside of *metV* and *metF* (Figure 1C). Subsequently, the absence of *metVF* in the chromosome was confirmed by DNA sequencing (Sanger et al., 1977).

### Growth phenotype of the ΔmetVF mutant

The mutant did not grow on H_2_ + CO_2_, formate, methanol or glycine betaine, demonstrating that the WLP is essential for C1 metabolism. The mutant also did not grow on ethanol, 2,3‐butanediol, acetoin or ethylene glycol, demonstrating the essentiality of the WLP as electron sink for the oxidation of alcohols. However, the situation was different with substrates that are metabolised via pyruvate. Cells grew on pyruvate and dihydroxyacetone but did not grow on lactate or alanine. Of special interest was the metabolism of hexoses such as fructose since these are common substrates for acetogens and the Δ*metVF* mutant was obtained on fructose‐containing agar plates.

**FIGURE 2:**
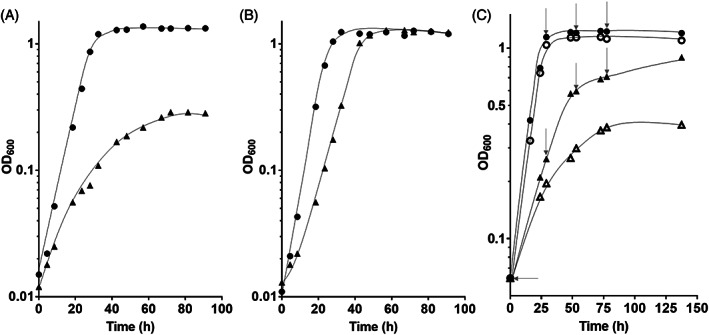
Growth of the Δ*metVF* mutant on fructose with alternative electron acceptors. Growth experiments were carried out in 5 mL complex medium (containing bicarbonate under a N_2_/CO_2_ atmosphere; 80:20, v/v) in 16 mL Hungate tubes at 30°C with (A) 20 mM fructose, (B) 20 mM fructose + 80 mM glycine betaine and (C) 20 mM fructose + 8 mM caffeate. Depicted are the optical densities of the wild‐type (●) and the Δ*metVF* mutant (▲). (C) 2 mM Caffeate was added at 0, 29, 53 and 78 h of incubation. Black, with caffeate; white, without caffeate. The growth experiments were performed in biological triplicates and one representative growth curve is presented.

The Δ*metVF* mutant grew on fructose but growth was poor with a growth rate of 0.05 h^−1^ and a final OD_600_ of only 0.3, which is only 26% and 10% of the wild‐type values (Figure 2A). During 90 h of incubation, only 7.1 ± 0.5 mM of fructose was consumed, and only 8.9 ± 0.0 mM of acetate was produced with a fructose:acetate ratio of 1:1.25. This implies that acetate was not formed via the WLP, instead, 8.1 ± 1.9 mM of H_2_, 6.6 ± 0.2 mM of formate, 1.0 ± 0.2 mM of ethanol and 2.1 ± 0.3 mM of lactate were produced.

### Alternative electron acceptors restore growth of the ΔmetVF mutant on fructose to wild‐type levels

Apparently, the Δ*metVF* mutant did grow on fructose, but growth was rather poor. If this phenotype was due to the loss of an efficient pathway that enables reoxidation of reduced electron carriers, that is, the WLP, growth should be restored by addition of other electron acceptors. The substrates that feed carbon into the pathway after methylene‐THF are methyl group‐containing substrates. Indeed, we did show very recently, that methanol + CO_2_ can be used as an electron sink; excess electrons are used to reduce CO_2_ to CO which is then condensed with the methyl group and CoA to acetyl‐CoA (Kremp et al., 2018; Litty et al., 2022). Here, we used glycine betaine as methyl group donor. The mutant did not grow on glycine betaine alone but in the presence of glycine betaine (plus CO_2_ present in the medium), growth on fructose was restored to wild‐type levels (Figure 2B). Ethanol, formate, lactate and H_2_ were no longer formed. Caffeate is another alternative electron acceptor that is reduced by *A. woodii* to hydrocaffeate (Dilling et al., 2007; Hess, Gonzalez, et al., 2013). Addition of caffeate to the culture grown on fructose fully restored growth to the wild‐type level and, again, formate, ethanol, lactate and H_2_ were no longer formed (Figure 2C). Apparently, production of short chain fatty acids, ethanol and H_2_ was turned off in the presence of an energetically more advantageous external electron acceptor.

### Transcriptome of fructose metabolism in the ΔmetVF mutant

Comparative genome‐wide expression profiling was performed with the wild‐type and the ∆*metVF* mutant during growth on fructose to address regulation of key enzymes and to shed light on the enzymes involved in formation of ethanol, lactate, formate and H_2_. There is only one hydrogenase present in *A. woodii* that can produce H_2_ from NADH and reduced ferredoxin, the electron‐bifurcating hydrogenase HydABC (Schuchmann & Müller, 2012). However, expression of the encoding genes (Awo_c26970–Awo_c27010) in the mutant did not change in a physiologically significant range compared to the wild‐type, but transcript counts were rather high in the mutant and the wild‐type (Table 1). Expression of the HDCR (Awo_c08190–Awo_c08260) also did not change to a physiologically significant extent, but the two iso‐genes encoding the formyl‐tetrahydrofolate synthetase (*fhs1*, Awo_c09260; *fhs2*, Awo_c08040) and the following genes of the WLP were upregulated 2‐ to 3.5‐fold. A gene encoding the potential formate transporter (Awo_c08050) was also upregulated 3.1‐fold. Energy conservation via Rnf complex and ATP synthase seemed not to be affected at the level of gene expression.

**TABLE 1:** Transcript abundance of genes encoding key enzymes for fructose metabolism in the ∆*metVF* mutant.

Gene	Annotation	Function	Transcript counts		Fold change (log_2_)
			∆*metVF*	Wild‐type	
Awo_c08190	Formate dehydrogenase FdhF1	HDCR	280	256	0.13
Awo_c08200	Hydrogenase, Fe–S subunit HycB1		38	44	−0.19
Awo_c08210	Formate dehydrogenase FdhF2		28,916	30,559	−0.08
Awo_c08230	Hydrogenase, Fe–S subunit HycB2		4994	3548	0.49
Awo_c08240	Formate dehydrogenase accessory protein FdhD		3718	3313	0.16
Awo_c08250	Hydrogenase, Fe–S subunit HycB3		2255	2188	0.04
Awo_c08260	Iron hydrogenase HydA2		7358	9540	−0.37
Awo_c08050	Formate/nitrite transporter FdhC	Formate transport	58,656	6795	3.1
Awo_c09260	Formyl‐THF synthetase Fhs1	Synthesis of formyl‐THF	133,326	32,132	2.05
Awo_c08040	Formyl‐THF synthetase Fhs2		413,872	35,848	3.52
Awo_c09270	Methenyltetrahydrofolate cyclohydrolase FchA	WLP methyl branch	29,762	5362	2.46
Awo_c09280	Methylenetetrahydrofolate dehydrogenase FolD	WLP methyl branch	48,052	11,292	2.08
Awo_c09290	Electron transport complex Rnf, C subunit RnfC2	MTHFR	171,346	29,713	2.52
Awo_c10670	CODH Ni^2+^‐insertion accessory protein CooC1	ACS/CODH	1000	1997	−0.99
Awo_c10680	Corrinoid activation/regeneration protein		4126	7952	−0.94
Awo_c10690	Hypothetical protein		1356	2178	−0.68
Awo_c10700	Hypothetical protein		933	1748	−0.9
Awo_c10710	CFeS protein, SSU AcsD		16,808	36,479	−1.11
Awo_c10720	CFeS protein, LSU AcsC		28,276	63,571	−1.16
Awo_c10730	Methyltransferase 2		14,878	20,910	−0.49
Awo_c10740	CODH, catalytic subunit AcsA		34,576	40,024	−0.2
Awo_c10750	CODH Ni^2+^‐insertion accessory protein CooC2		13,543	10,313	0.39
Awo_c10760	ACS, catalytic subunit AcsB		36,085	40,282	−0.15
Awo_c19620	Phosphotransacetylase Pta	Acetate formation	3950	2387	0.72
Awo_c21260	Acetate kinase AckA		3858	11,972	−1.63
Awo_c22010	Electron transport complex protein RnfB	RnF complex	3421	6917	−1.01
Awo_c22020	Electron transport complex protein RnfA		1227	3017	−1.29
Awo_c22030	Electron transport complex protein RnfE		1739	4216	−1.27
Awo_c22040	Electron transport complex protein RnfG		1666	4351	−1.38
Awo_c22050	Electron transport complex protein RnfD		2939	9086	−1.62
Awo_c22060	Electron transport complex protein RnfC1		4844	11,125	−1.19
Awo_c26970	Iron hydrogenase HydA1	Bifurcating hydrogenase	63,435	56,607	0.17
Awo_c26980	Iron hydrogenase HydB		68,393	49,927	0.45
Awo_c26990	Iron hydrogenase HydD		7064	5220	0.43
Awo_c27000	Sensory transduction histidine kinase HydE		12,305	8710	0.5
Awo_c27010	Iron hydrogenase HydC		4010	4073	−0.02
Awo_c02140	ATP synthase protein I AtpI	ATP synthase	267	893	−1.73
Awo_c02150	F‐type ATP synthase subunit A AtpB		5603	14,149	−1.32
Awo_c02160	F‐type ATP synthase subunit E AtpE1		2118	4632	−1.1
Awo_c02170	F‐type ATP synthase subunit E AtpE2		791	1668	−1.06
Awo_c02180	F‐type ATP synthase subunit E AtpE3		566	1629	−1.51
Awo_c02190	F‐type ATP synthase subunit F AtpF		2300	4399	−0.92
Awo_c02200	F‐type ATP synthase subunit H AtpH		2754	5879	−1.08
Awo_c02210	F‐type ATP synthase subunit A AtpA		19,547	45,557	−1.21
Awo_c02220	F‐type ATP synthase subunit G AtpG		8197	13,349	−0.69
Awo_c02230	F‐type ATP synthase subunit D AtpD		19,638	39,837	−1.01
Awo_c02240	F‐type ATP synthase subunit C AtpC		1623	2043	−0.32
Awo_c01690	Dihydrolipoamide dehydrogenase	Acetoin metabolism	635,893	1013	**9.28**
Awo_c01700	Pyruvate dehydrogenase E2 component (dihydrolipoamide acetyltransferase)		623,205	1442	**8.75**
Awo_c01710	Pyruvate dehydrogenase E1 component beta subunit		638,263	1586	**8.61**
Awo_c01720	Pyruvate dehydrogenase E1 component alpha subunit		689,472	2744	**7.93**
Awo_c01730	Transcriptional regulator of acetoin/glycerol metabolism		2799	2101	0.40
Awo_c08710	Electron transfer flavoprotein beta subunit	Lactate metabolism	76,533	548	**7.12**
Awo_c08720	Electron transfer flavoprotein alpha subunit		123,511	505	**7.93**
Awo_c08730	Lactate dehydrogenase		226,503	653	**8.44**
Awo_c08740	Lactate permease		93,321	346	**8.08**
Awo_c08750	Lactate racemase		71,991	315	**7.84**
Awo_c12730	CxxC motif‐containing protein	Glycerol metabolism	18,012	2346	2.92
Awo_c12740	Thioredoxin reductase		69,136	8924	2.93
Awo_c12750	Glycerol‐3‐phosphate dehydrogenase		82,464	11,424	2.84
Awo_c12760	Glycerol uptake operon antiterminator		11,904	2880	2.03
Awo_c12770	Glycerol kinase		154,543	43,460	1.82
Awo_c15700	Propionate CoA‐transferase CarA	Caffeate metabolism	1041	54	4.27
Awo_c15710	Fatty‐acyl‐CoA synthase CarB		1188	46	4.69
Awo_c15720	Acyl‐CoA dehydrogenase CarC		1006	59	4.09
Awo_c15730	Electron transfer flavoprotein beta subunit CarD		1220	52	4.55
Awo_c15740	Electron transfer flavoprotein alpha subunit apoprotein CarE		2209	125	4.14
Awo_c24340	Alanine or glycine:cation symporter	Alanine metabolism	453	123	1.84
Awo_c24350	Alanine dehydrogenase		9213	863	3.39
Awo_c24360	AsnC family transcriptional regulator AldR		1660	218	2.91
Awo_c25770	CoA‐dependent propionaldehyde dehydrogenase PduP	Alcohol metabolism	2647	12,013	−2.17
Awo_c33720	Aldehyde dehydrogenase Aldh		523	963	−0.61
Awo_c04510	Alcohol dehydrogenase Adh1		296	259	0.19
Awo_c05670	Alcohol dehydrogenase Adh2		1241	866	0.51
Awo_c06180	Alcohol dehydrogenase Adh3		2185	1747	0.31
Awo_c06220	Alcohol dehydrogenase Adh4		1250	2717	−1.09
Awo_c06310	Bifunctional acetaldehyde‐CoA/alcohol dehydrogenase		371	413	−0.15
Awo_c14890	Alcohol dehydrogenase Adh5		26	16	0.59
Awo_c25410	Alcohol dehydrogenase Adh6		737	1827	−1.30
Awo_c26370	Alcohol dehydrogenase Adh7		408	651	−0.66
Awo_c00730	Butanol dehydrogenase Bdh1		661	882	−0.40
Awo_c02950	Butanol dehydrogenase Bdh2		1564	1522	0.04
Awo_c17360	1,3‐propanediol dehydrogenase dhaT		67	22	1.56

*Note*: Bold values: log_2_ fold change over +6.0.

Abbreviations: ACS, acetyl‐CoA synthase; CODH, CO dehydrogenase.

*A. woodii* encodes an electron‐bifurcating lactate dehydrogenase‐Etf‐complex (Weghoff et al., 2015) but apparently no other lactate dehydrogenase. The transcript levels of these genes together with the genes encoding a lactate racemase and a lactate permease (Awo_c08710–Awo_c08750) were highly upregulated (7.1‐ to 8.4‐fold) in the mutant compared to the wild‐type. Ethanol can be formed from acetyl‐CoA by CoA‐dependent acetaldehyde dehydrogenases and alcohol dehydrogenases. *A. woodii* contains several genes encoding CoA‐dependent acetaldehyde dehydrogenases and alcohol dehydrogenases, but the transcriptome data did not reveal a striking differential regulation of any of these genes. However, transcription of a gene cluster encoding a potential pyruvate dehydrogenase/acetoin dehydrogenase (Awo_c01690–Awo_c01720) showed the highest level of upregulation in our studies.

### Acetate and hydrogen formation from fructose in resting cells of the ΔmetVF mutant grown on fructose + glycine betaine

**FIGURE 3:**
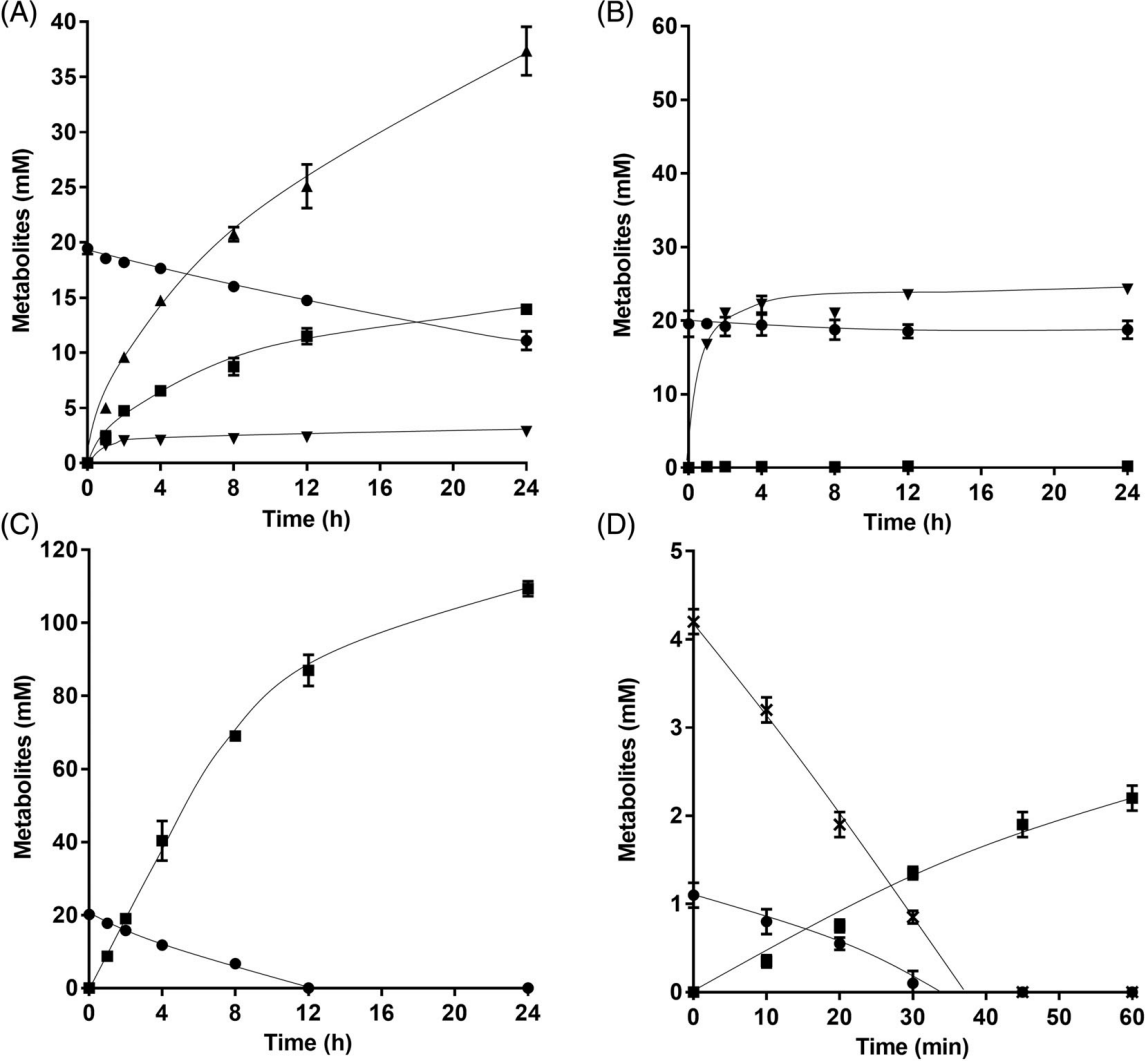
Conversion of fructose in resting cells of the Δ*metVF* mutant. Cells of the Δ*metVF* mutant were grown in bicarbonate‐buffered complex media under a N_2_/CO_2_ atmosphere (80:20, v/v) with 20 mM fructose + 80 mM glycine betaine and harvested in the early stationary growth phase. After washing, the cells were resuspended in 10 mL of cell suspension buffer (50 mM imidazole, 20 mM MgSO_4_, 20 mM KCl, 20 mM NaCl, 60 mM KHCO_3_, pH 7.0) in 120 mL serum flasks under a N_2_/CO_2_ atmosphere at a total protein concentration of 2 mg/mL. (A) 20 mM fructose, (B) 20 mM fructose + H_2_, (C) 20 mM fructose + 80 mM glycine betaine was given to resting cells as carbon and energy source. (D) For conversion of 1 mM fructose + 4 mM caffeate, resting cells were prepared from the mutant grown on 20 mM fructose + 8 mM caffeate. Fructose (●), acetate (■), H_2_ (▲), formate (▼) and caffeate (×) were determined. Each data point indicates a mean with standard error of the mean; *n* = 2 independent experiments.

In cells of the Δ*metVF* mutant growing on fructose, metabolism was shifted towards reduced end products due to the block in the WLP. Fructose metabolism of the Δ*metVF* mutant was further investigated in resting cells to analyse catabolic electron flow only. For these experiments, cells were grown on 20 mM of fructose + 80 mM of glycine betaine until stationary growth phase, harvested, washed and resuspended in 10 mL of imidazole buffer as described in Experimental Procedures. When 20 mM of fructose was given to the resting cells of the Δ*metVF* mutant, only 14.0 ± 0.2 mM of acetate was produced from 9.1 ± 0.7 mM with a fructose:acetate ratio of 1:1.5 (Figure 3A). A minor amount of formate (2.9 ± 0.2 mM) was produced but lactate and ethanol were not produced. Much to our surprise, H_2_ was produced in huge amounts with 37.4 ± 2.2 mM, giving a fructose:H_2_ ratio of 1:4.1. This is the maximum amount of H_2_ that can be produced from fructose (see Equation 5). When H_2_ was given in addition to fructose, fructose was no longer consumed, and acetate was not formed, indicating an inhibition of fructose oxidation by molecular hydrogen. However, formate was accumulated to high concentrations (24.3 ± 0.4 mM; Figure 3B) under these conditions, indicating that the HDCR converts bicarbonate/CO_2_ (present in the buffer) with H_2_ to formate with high rates, as observed previously (Kottenhahn et al., 2018; Schuchmann & Müller, 2013). When a methyl group (80 mM of glycine betaine) was added as electron sink (together with CO_2_/bicarbonate present in the medium), fructose consumption accelerated and was 6.6 times faster than in the absence of glycine betaine (Figure 3C). Resting cells performed homoacetogenesis and produced 109.4 ± 2.1 mM of acetate with a fructose:acetate ratio of 1:5.4. Hydrogen was no longer produced in the presence of glycine betaine. Addition of the alternative electron sink caffeate to cells metabolising fructose also increased fructose consumption rates and completely abolished H_2_ production; the reducing equivalents were apparently used to reduce caffeate to hydrocaffeate (Figure 3D). 2.2 ± 0.1 mM of acetate was formed from 1.1 ± 0.1 mM of fructose in the presence of 4.2 ± 0.1 mM of caffeate with a fructose:acetate ratio of 1:2.

### MAF with fructose in resting cells of the ΔmetVF mutant grown on fructose

**FIGURE 4:**
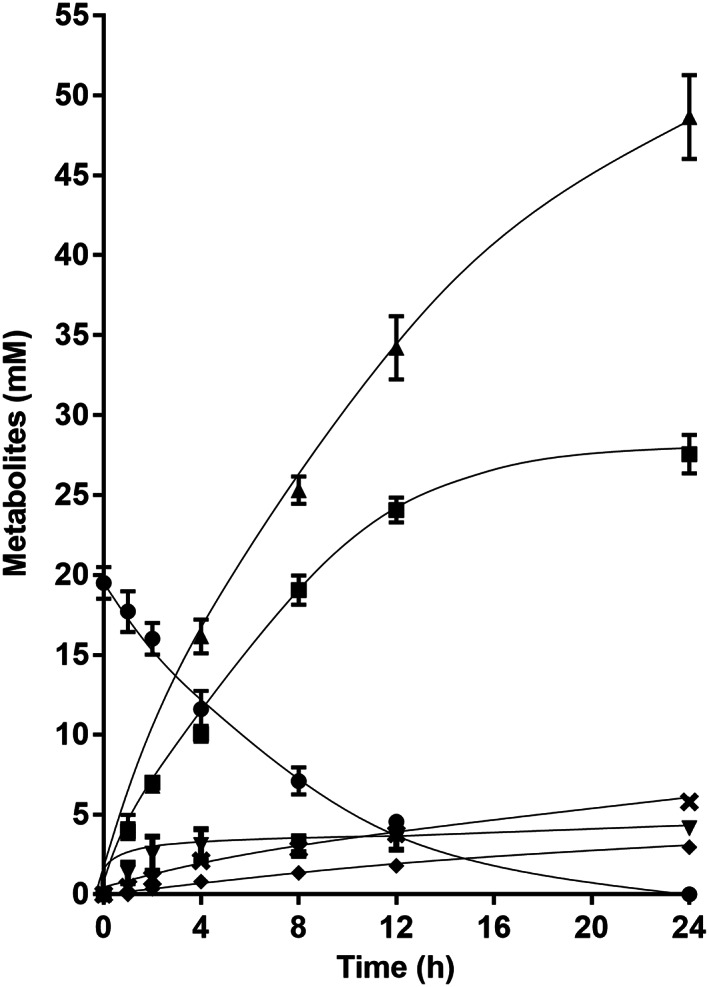
Fermentation in fructose‐adapted resting cells of the Δ*metVF* mutant. Cells of the Δ*metVF* mutant were grown in bicarbonate‐buffered complex media under a N_2_/CO_2_ atmosphere (80:20, v/v) with 20 mM fructose and harvested in the stationary growth phase (OD_600_ of 0.3). After washing, the cells were resuspended in 10 mL of cell suspension buffer (50 mM imidazole, 20 mM MgSO_4_, 20 mM KCl, 20 mM NaCl, 60 mM KHCO_3_, pH 7.0) in 120 mL serum flasks under a N_2_/CO_2_ atmosphere at a total protein concentration of 2 mg/mL. 20 mM fructose was given to resting cells as carbon and energy source. Fructose (●), acetate (■), H_2_ (▲), formate (▼), ethanol (♦) and lactate (×) were determined at each time point. Each data point indicates a mean ± SEM; *n* = 2 independent experiments.

Whereas growing cells produced hydrogen as well as formate, lactate and ethanol in addition to acetate, resting cells described earlier did not. However, the cells used for these experiments were pre‐grown on fructose + glycine betaine, conditions under which formate, lactate and ethanol were not formed, opening the possibility that the ability to produce formate, lactate and ethanol was turned off by the presence of glycine betaine as electron sink in the growth medium. To check this possibility, we grew cells on fructose only, prepared resting cells and analysed product formation from fructose. These cells converted 19.5 ± 1.0 mM of fructose to 27.6 ± 1.2 mM of acetate with a fructose:acetate ratio of 1:1.41 (Figure 4). However, as in growing cells, we found formate (4.2 ± 0.2 mM), ethanol (3.0 ± 0.2 mM) and lactate (5.8 ± 0.3 mM) along with hydrogen (48.7 ± 2.6 mM). Acetoin, glycerol and alanine were not produced. As observed in growing cells, intermediates (pyruvate and acetyl‐CoA) as well as end products (CO_2_) were reduced under these conditions, demonstrating the ability of *A. woodii* for a MAF of fructose.

## DISCUSSION

The metabolism of acetogenic bacteria shows an extraordinary diversity. They can grow organotrophically as well as lithotrophically; central to both types of metabolism is the WLP. It allows growth on C1 compounds such as H_2_ + CO_2_, formate, methyl groups and carbon monoxide (Diekert & Thauer, 1978; Genthner & Bryant, 1982; Kremp et al., 2018; Kremp & Müller, 2021; Lechtenfeld et al., 2018; Litty et al., 2022; Moon et al., 2021; Stupperich & Konle, 1993; van der Meijden et al., 1984; Weghoff & Müller, 2016; Wood et al., 1986). The WLP allows to conserve energy, either by the pathway itself, for example the formyl‐THF synthetase in the oxidative reaction during methyl group oxidation (Kremp et al., 2018; Kremp & Müller, 2021; Lechtenfeld et al., 2018; Litty et al., 2022; Moon et al., 2023), or by a respiratory chain involved in redox balancing (Hess, Schuchmann, & Müller, 2013). However, the amount of ATP generated is small compared to the amount of ATP generated by glycolysis and the acetate kinase reaction. The use of the external electron acceptor CO_2_ allows acetogenic bacteria that grow on hexoses to produce the maximum amount of ATP that can be obtained by hexose fermentation. Interestingly, many acetogens can use alternative electron acceptors such as nitrate, dimethylsulfoxide, fumarate or even phenylacrylates (Bache & Pfennig, 1981; Matthies et al., 1993; Rosenbaum et al., 2022; Seifritz et al., 1993). Here, we have identified another electron acceptor previously unknown for *A. woodii* and other mesophilic acetogens, protons. Apparently, *A. woodii* converted fructose and the reducing equivalents were released as H_2_. Hydrogenogenesis has been found before in several anaerobes (Schröder et al., 1994; O‐Thong et al., 2008; Pradhan et al., 2015) and among them so far only one thermophilic acetogen, *T. kivui* (Moon et al., 2020). Hydrogenogenesis requires hydrogen formation from the reduced electron carriers involved in acetogenesis from fructose in *A. woodii* which are NADH and reduced ferredoxin. Hydrogen formation from NADH is thermodynamically restricted but made possible in *A. woodii* by the electron‐bifurcating hydrogenase HydABC that catalyses the following reaction (Schuchmann & Müller, 2012, 2014):

(6) $$\begin{array}{l} {\text{3H}}^{+}+\text{NADH}+{\mathrm{Fd}}^{2-}\\ \;\to {\text{2H}}_{2}+{\mathrm{NAD}}^{+}+\mathrm{Fd}\;\text{∆}{G_{0}}^{\prime }=+11\;\mathrm{kJ}/\mathrm{mol}. \end{array}$$

Hydrogenogenesis is also the preferred way of metabolism in co‐culture of *A. woodii* with a methanogen (Heijthuijsen & Hansen, 1986; Winter & Wolfe, 1980). Under these conditions, the methanogen keeps the hydrogen concentration low, making hydrogen formation according to Equation 6 thermodynamically more favourable. Of course, high concentrations of the end product, H_2_, inhibit further hydrogen formation. This was observed in the presence of fructose + H_2_ in *A. woodii* that inhibited further hydrogen formation and thus, fructose oxidation.

**FIGURE 5:**
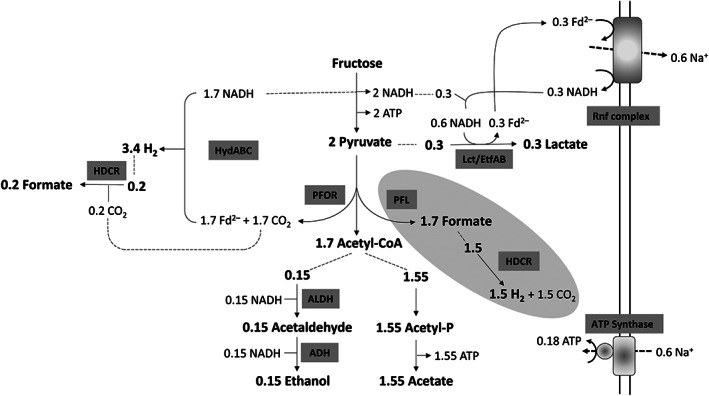
Biochemistry and bioenergetics of mixed acid fermentation from fructose in the Δ*metVF* mutant of *Acetobacterium woodii*. ADH, alcohol dehydrogenase; ALDH, aldehyde dehydrogenase; Fd, ferredoxin; HDCR, H_2_‐dependent CO_2_ reductase; HydABC, electron‐bifurcating hydrogenase; Lct/EtfAB, electron‐bifurcating lactate dehydrogenase; PFL, pyruvate:formate lyase; PFOR, pyruvate:ferredoxin oxidoreductase. The stoichiometry of the ATP synthase is 3.3 Na^+^/ATP (Matthies et al., 2014) and for the Rnf complex a stoichiometry of 2 Na^+^/2 e^−^ is assumed. The involvement of the PFL pathway (in grey) seems unlikely. For further explanations, see text.

In the absence of a suitable electron acceptor such as CO_2_ or when the thermodynamics does not allow for hydrogen formation, *A. woodii* as well as *T. kivui* did not grow (Jain et al., 2020; Moon et al., 2023). This has always been puzzling since at least *A. woodii* has the genetic potential for MAF. Indeed, here we did observe MAF from fructose for the first time in the *metVF* mutant. *A. woodii* has three copies of a pyruvate:formate lyase gene and thus, formate may have been produced by the PFL (Transcript levels of *pfl* genes were upregulated 0.1–1.5 fold). The formate produced may then have been converted to H_2_ and CO_2_ by the HDCR enzyme. A quantitative model of MAF that reflects the concentrations of the products formed, is shown in Figure 5. If we take 0.3 mol of pyruvate for lactate formation, as determined experimentally, that leaves 1.7 mol of acetyl‐CoA. 0.15 mol are reduced to ethanol (experimentally determined: 0.15 mol of ethanol per mol of fructose), which leaves 1.55 mol of acetyl‐CoA that can be oxidised to acetate (experimentally determined: 1.41 mol of acetate per mol of fructose). Pyruvate oxidation by PFL yields 1.7 mol of formate. Since only 0.2 mol of formate per mol of fructose were found, 1.5 could have been converted to 1.5 mol of H_2_ and 1.5 mol of CO_2_. However, not 1.5 mol of H_2_ but 2.5 were experimentally determined. In addition, 1.7 mol of NADH are left over from glycolysis in this model. Hydrogen production from NADH requires reduced ferredoxin but the only way to get reduced ferredoxin is via the PFOR reaction. If we assume 1.7 mol of pyruvate to be oxidised by PFOR instead of PFL, this would yield 1.7 reduced ferredoxin that together with 1.7 mol of NADH would give 3.4 mol of H_2_. Therefore, we assume the PFL way to be unlikely but further genetic analyses will shed light on the role of the PFL. The situation is even more complicated in light of the three potential *pfl* genes (Poehlein et al., 2012).

Here, production of lactate as an end product has been observed for the first time in *A. woodii*. Since *A. woodii* has only one lactate dehydrogenase, the electron‐bifurcating Ldh/Etf complex, reduction of pyruvate is accompanied with the reduction of ferredoxin (Weghoff et al., 2015). Reoxidation of reduced ferredoxin with concomitant reduction of NAD by the Rnf complex conserves additional energy by a chemiosmotic mechanism (Hess, Schuchmann, & Müller, 2013). Ethanol formation could proceed via acetaldehyde to ethanol by aldehyde dehydrogenase ALDH/PduP plus alcohol dehydrogenase Adh4/6 (Moon & Müller, 2021).

In sum, we have demonstrated MAF from fructose in *A. woodii* when the WLP has been genetically inactivated. Genes encoding enzymes for the production of lactate, ethanol, formate and hydrogen are present in *A. woodii*, but their role in this new metabolic trait of *A. woodii* have to be determined by genetic and biochemical assays in the near future.

## AUTHOR CONTRIBUTIONS

**Jimyung Moon:** Conceptualization (equal); investigation (lead); methodology (lead); project administration (lead); visualization (lead); writing – original draft (equal); writing – review and editing (equal). **Anja Schubert:** Investigation (supporting); resources (supporting). **Anja Poehlein:** Data curation (supporting); formal analysis (supporting); resources (supporting). **Rolf Daniel:** Supervision (supporting). **Volker Müller:** Conceptualization (equal); funding acquisition (lead); resources (lead); supervision (lead); writing – original draft (equal); writing – review and editing (equal).

## CONFLICT OF INTEREST STATEMENT

The authors declare that there is no conflict of interest.

## Data Availability

The data generated in this study are available from the corresponding author on reasonable request.
